# Supplementary material for: Liver-Targeted Nanoparticles Facilitate the Bioavailability and Anti-HBV Efficacy of Baicalin In Vitro and In Vivo
Source: Biomedicines. 2022 Apr 14;10(4):900. doi: 10.3390/biomedicines10040900 (PMC9025464; doi:10.3390/biomedicines10040900)
Supplement: Supplementary file 1 [file biomedicines-10-00900-s001.zip › biomedicines-1640656-supplementary.pdf]

Supplement Materials

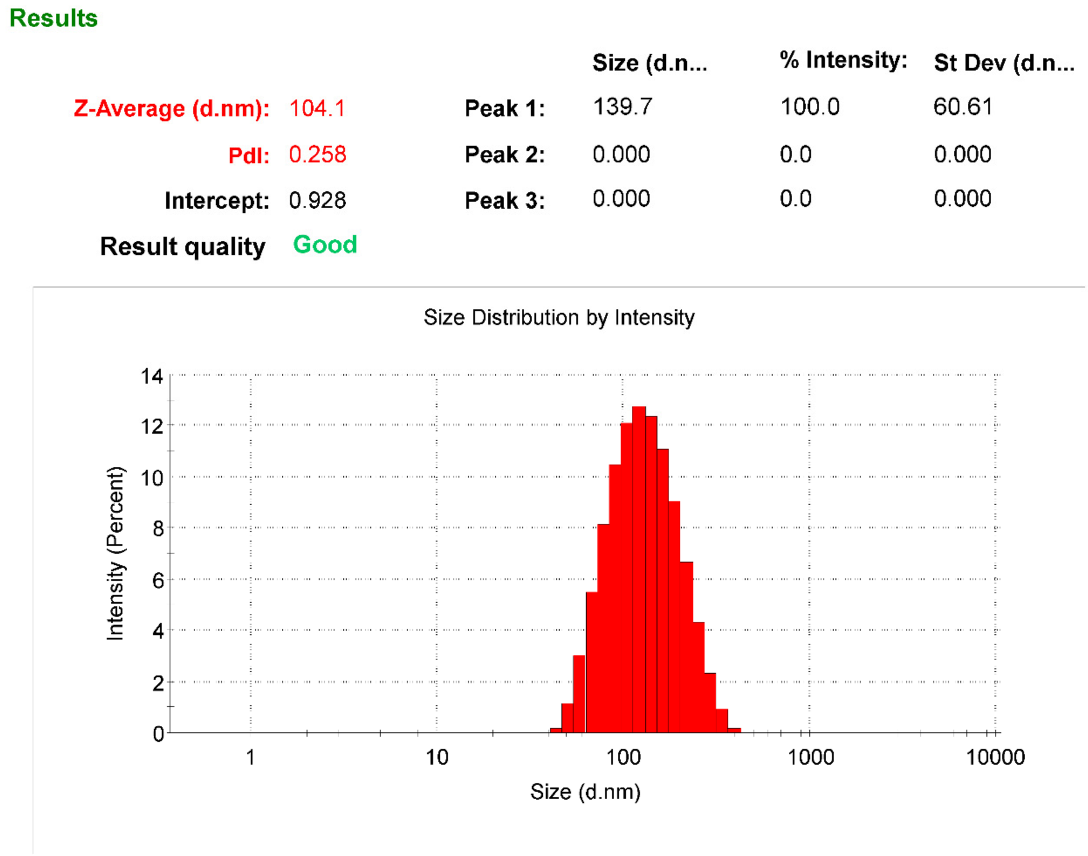

Figure S1. PDI and particle size of BAA1 were detected by the Malvern Zetasizer Nano ZS90.

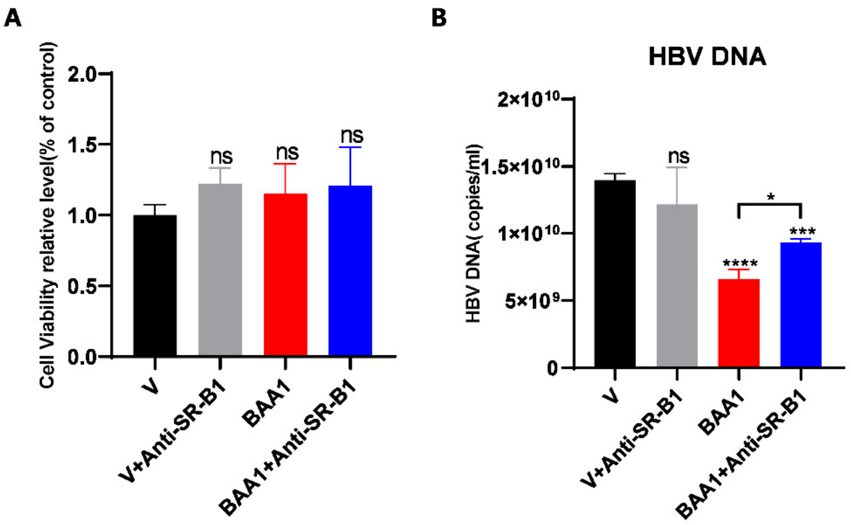

Figure S2. HepG2 cells were transfected with pHBV1.2 followed by treatment with BAA1 (50 µM) or anti-SR-B1 (1:100, *v/v*) for 2 days. (**A**) The cell viability was

obtained according to the standard protocol of Cell Counting Kit-8 (Beyotime Biotechnology, Shanghai, China). (**B**) HBV DNA was detected by qRT-PCR. Data are mean ± SD, n = 3; * $p < 0.05$, *** $p < 0.001$ and **** $p < 0.0001$ vs. Control. ns, not significant.

Table S1. Primers used for qRT-PCR.

**Primer Name**	**Sequence（5′ –3′ ）**	
HNF1α	Forward	CCTGTCCCAACACCTCAACAA
	Reverse	TTGAAACGGTTCCTCCGC
FOXA2	Forward	AGGAGGAAAACGGGAAAGAA
	Reverse	CTGCAACAACAGCAATGGAG
HNF4α	Forward	GGAGCTGGCGGAGATGAGCC
	Reverse	CGCGAGTCATACTGGCGGTCG
pgRNA	Forward	CTCAATCTCGGGAATCTCAATGT
	Reverse	TGGATAAAACCTAGCAGGCATAAT
Total HBV-specific transcripts	Forward	ATCCTGCTGCTATGCCTCATCTT
	Reverse	ACAGTGGGGGAAAGCCCTACGAA
HBV-DNA	Forward	TCACCAGCACCATGCAAC
	Reverse	AAGCCACCCAAGGCACAG
hB1F	Forward	GGCTTATGTGCAAAATGGCAGATC
	Reverse	GCTCACTCCAGCAGTTCTGAAG
PS2	Forward	CCAGGCCCAGGAAGAAACAT
	Reverse	AACAGCAACCTCTCTCCGTG
GAPDH	Forward	CATGTTCGTCATGGGGTGAACCA
	Reverse	AGTGATGGCATGGACTGTGGTCAT
